# Gas sensing properties of MWCNT layers electrochemically decorated with Au and Pd nanoparticles

**DOI:** 10.3762/bjnano.8.64

**Published:** 2017-03-10

**Authors:** Elena Dilonardo, Michele Penza, Marco Alvisi, Riccardo Rossi, Gennaro Cassano, Cinzia Di Franco, Francesco Palmisano, Luisa Torsi, Nicola Cioffi

**Affiliations:** 1 Department of Chemistry, Università degli Studi di Bari Aldo Moro, Bari, Italy; 2 Department of Electrotechnics and Electronics, Politecnico di Bari, Bari, Italy; 3 Italian National Agency for New Technologies, Energy and Sustainable Economic Development (ENEA), Laboratory Functional Materials and Technologies for Sustainable Applications - Brindisi Research Center, Brindisi, Italy,; 4 CNR-IFN Bari, Bari, Italy

**Keywords:** Au nanoparticle, chemiresistive gas sensor, electrophoretic deposition, electrosynthesis, metal-functionalized MWCNTs, Pd nanoparticle

## Abstract

Multiwalled carbon nanotube (MWCNT)-based chemiresistors were electrochemically decorated with Au and Pd nanoparticles (NPs), resulting in an improvement in the detection of gaseous pollutants as compared to sensors based on pristine MWCNTs. Electrophoresis was used to decorate MWCNTs with preformed Au or Pd NPs, thus preserving their nanometer-sized dimensions and allowing the metal content to be tuned by simply varying the deposition time. The sensing response of unmodified and metal-decorated MWCNTs was evaluated towards different gaseous pollutants (e.g., NO_2_, H_2_S, NH_3_ and C_4_H_10_) at a wide range of concentrations in the operating temperature range of 45–200 °C. The gas sensing results were related to the presence, type and loading of metal NPs used in the MWCNT functionalization. Compared to pristine MWCNTs, metal-decorated MWCNTs revealed a higher gas sensitivity, a faster response, a better stability, reversibility and repeatability, and a low detection limit, where all of these sensing properties were controlled by the type and loading of the deposited metal catalytic NPs. Specifically, in the NO_2_ gas sensing experiments, MWCNTs decorated with the lowest Au content revealed the highest sensitivity at 150 °C, while MWCNTs with the highest Pd loading showed the highest sensitivity when operated at 100 °C. Finally, considering the reported gas sensing results, sensing mechanisms have been proposed, correlating the chemical composition and gas sensing responses.

## Introduction

Today one of the trending worldwide issues is environmental pollution. Human activity, industries, transportation and agriculture, and natural phenomena, like volcano activity, are known to cause an increase of emissions of toxic, pollutant gases (e.g., NO_2_, H_2_S, NH_3_, C_4_H_10_) and greenhouse gases into the atmosphere [1]. The effect of high concentration levels of these gaseous pollutants causes both environmental problems and health consequences for humans (e.g., respiratory and cardiovascular illness). Hence, control and monitoring of gas emissions is still a critical need of modern societies.

So far several restrictions have been imposed to limit and to control the emissions of pollutant gases into the atmosphere, although their levels in the atmosphere are still kept below the environmental quality standards established by Kyoto and Paris climate agreements [2–3].

Besides the expensive and time-consuming analytical instrumentation (e.g., gas chromatography) commonly used to control the concentration of released gases, some progress has been made in gas sensor technologies as a valid alternative in gas detection. Indeed, gas sensor systems have unique properties such as, easy and low-cost production, low operating temperature, electronic and thermal stability, good sensitivity but poor selectivity in gas detection [4]. In this context, the development of nanostructured materials has contributed greatly to the improvement and diffusion of gas sensor technology [5]. Specifically, the discovery of carbon nanotubes (CNTs) has extensively advanced gas sensor applications [6]. Indeed, CNTs are a class of promising materials in the field of gas sensors, thanks to their unique properties, such as a large surface area and hollow structure, that make them potentially applicable as a sensing layer in gas sensors [7–8]. Semiconducting MWCNTs are frequently used in chemiresistors, since they are extremely sensitive to charge transfer and chemical doping effects in the presence of oxidizing or reducing analytes, reveling mostly a p-type behavior [9–10].

Recently, researches have revealed not only the positive properties of CNT-based gas sensors, but also their weaknesses: weak sensing response with low selectivity and long recovery. These disadvantages are caused by strong interactions between the analyte gaseous molecules and CNTs, making their desorption difficult [11–12].

Several attempts have been made to improve the CNT-based sensor performance by modifying CNTs with polymeric composites [13], and/or catalyst metals, hybrid, and other catalytic materials [14]. Recently, a surfactant-free approach, not requiring any post-treatment for the removal of dispersants or any CNT functionalization, has been developed to reduce the production costs and, at the same time, to obtain CNTs with improved sensing performance thanks to the use of continuous in situ UV irradiation to accelerate the gas desorption [15]. Beside these promising results, it is already well known that the modification of CNTs with selected materials can improve the sensitivity and selectivity of CNT-based sensors for several harmful gases [16].

The aim of decorating carbon nanotubes with metal nanoparticles (NPs) is to accelerate the surface reaction and the electron exchange between analyte and CNTs. Moreover, small gas molecules can strongly bond to transition metals thanks to their electronic structure and empty orbitals [17]. Therefore, CNTs decorated with metal NPs can improve the electrical properties and sensitivity of CNT-based sensors, due to an increase in the number of the adsorption sites for the targeted gases [18]. CNT sidewalls can be decorated with various noble metals such as Au, Pt, Pd, Rh, and Ag [19–24].

Different methods have been used to decorate CNTs, including electrodeposition [25–27], thermal evaporation [27–28], and sputtering [29–31]. Frequently, these deposition techniques result in an uncontrolled clustering at high metal loading, thus negatively affecting the catalytic properties and integrity of the carbon structure. As a possible solution to overcome these limits, we have proposed an electrophoretic process to directly deposit pre-synthesized colloidal gold NPs of controlled size and loading on the surface of MWCNT-based gas sensor devices [19]. Moreover, we have demonstrated that the MWCNT sensing properties of functionalized MWCNTs depend on the total surface amount of deposited Au NPs, and therefore, on the surface chemical composition.

In this contribution, we compare the sensing performance of Pd-modified MWCNT-based chemiresistors to that of Au-modified MWCNT-based chemiresistors (already investigated in previous study [19]) towards the detection of the oxidizing NO_2_ gas, and its interfering reducing gases, H_2_S, NH_3_. Different from the previous studies, here we extended the set of target species to butane (C_4_H_10_) gas, and we also explored a wider operating temperature range (45–200 °C).

Under these conditions, the effects of the presence of Pd and of its loading on the gas sensing performance of MWCNT-based chemiresistors were evaluated and critically compared to those of previously studied systems consisting of Au-decorated MWCNTs. The results confirm the improvement of the gas sensing performance of Pd-functionalized MWCNTs compared to the pristine material, and the influence of the deposited metal on the gas sensing results. Moreover, the sensing conditions (e.g., operating temperature) were varied for each hybrid system, and were shown to affect the sensing performance of pristine and decorated MWCNTs. Finally, based on the interplay between the surface chemical composition and gas sensing properties of hybrid gas sensors, sensing mechanisms have been proposed.

The electrophoretic process is herein shown to be a versatile and simple method to deposit various pre-synthesized colloidal metal NPs, specifically Au and Pd NPs, on MWCNTs, for gas sensing applications.

## Experimental

### Preparation of metal-decorated MWCNT-based chemiresistors

MWCNT networked films were grown by chemical vapor deposition (CVD) directly onto the surface of an alumina substrate that was previously coated with a cobalt (Co) sputtered catalytic layer (≈6 nm thick), as previously reported [32].

The two electrodes of the two-pole chemiresistors were made of Cr/Au (20 nm/300 nm) metal strips (vacuum sputtered onto MWCNT films), which were 1 mm in width and 5 mm in length with a 3 mm gap between the electrodes.

The Au or Pd NP colloidal solutions used to directly decorate MWCNT-based gas sensors were prepared by the sacrificial anode electrolysis (SAE) method, as reported elsewhere [33–34]. The electrochemical synthesis was carried out with a three-electrode cell consisting of an Ag/AgNO_3_ (0.1 M in acetonitrile) electrode, used as reference, and the metal, a gold or palladium sacrificial anode, used as the working electrode. A platinum cathode was used as the counter electrode. The electrolyte solution was composed of quaternary ammonium halide (0.05 M) dissolved in a 3:1 mixture of tetrahydrofuran and acetonitrile. Specifically, the quaternary ammonium salt was used both as a supporting electrolyte and as a nanoparticle capping agent. Tetrabutylammonium bromide (TBAB) was used for Pd NP synthesis and tetraoctylammonium chloride (TOAC) for the Au NPs [35].

**Figure 1:**
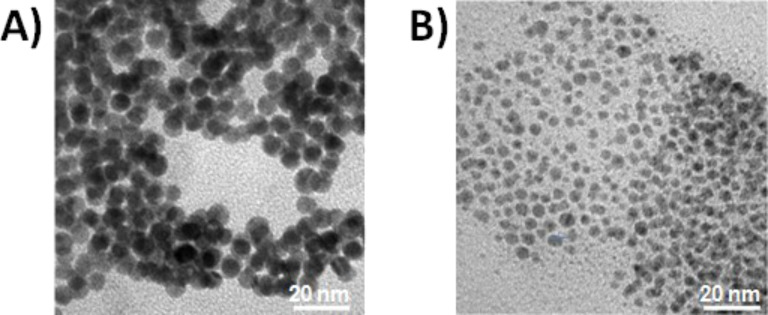
TEM images of electrochemically synthesized core–shell A) Au NPs and B) Pd NPs.

The electrochemically synthesized Au and Pd NPs had a uniform dispersion with a diameter of 12 ± 2 nm and 5.0 ± 0.5 nm, respectively, as reported in the TEM images in Figure 1.

The net positive surface charge of colloidal metal NPs, given by the quaternary ammonium surfactant [36–37], was used to functionalize the surface of the MWCNTs by electrophoresis. The lipophilic interactions between the alkyl group of the surfactant and MWCNTs assured the anchorage of the colloidal metal NPs on the surface of the MWCNTs [38].

MWCNT-based chemiresistors were functionalized by electrophoresis based on a three-electrode cell in which a Pt wire was the counter electrode. The MWCNT-based device was used as the working electrode and the Ag/AgNO_3_ (0.1 M in acetonitrile) system was the reference electrode [19]. The colloidal solution, consisting of 10 mM Au NPs or Pd NPs, and 5 mM tetraalkylammonium salt in tetrahydrofurane and acetonitrile mixture in 3:1 ratio, was the electrolytic solution. The electrophoretic deposition was a cathodic process in which the applied working potential was a little bit more negative than the open circuit potential (η = −200 mV), in order to induce the migration of the positively charged colloidal NPs towards the MWCNT-based device, used as cathode. This mild experimental approach prevented damage to the MWCNTs and, on the other hand, contributed to reduce (and remove) residual gold ions [39], resulting in the MWCNT surface decoration by metal NPs. Specificallly, the measured open circuit potential was −300 mV versus the reference electrode and the applied potential was −500 mV.

**Figure 2:**
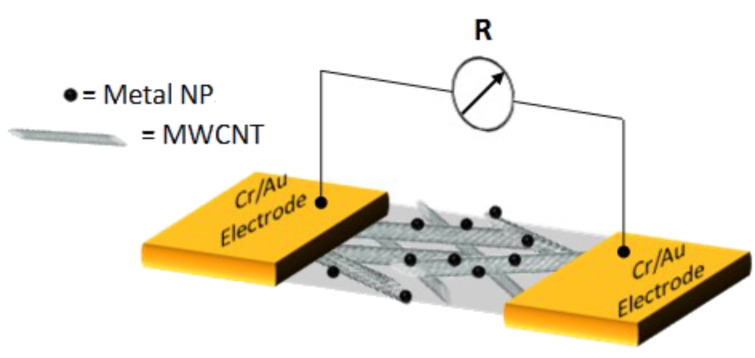
Schematic view of the two-pole chemiresistor based on a MWCNT network functionalized with metal NPs.

Finally, the residual surfactant was completely removed from the functionalized device by extensively washing it with acetonitrile three times. The process was performed using two different deposition times, 90 s and 600 s, resulting in different metal loadings. The scheme of the metal-functionalized MWCNT-based chemiresistive gas sensor device is reported in Figure 2.

### Material characterization

The surface chemical analysis was performed by using a Thermo VG Theta Probe XPS spectrometer with a μ-spot monochromatic Al Kα source. A fixed analyzer transmission mode with a pass energy of 150 eV was used to acquire XPS survey spectra; a pass energy of 100 eV was used for the acquisition of XPS high-resolution spectra.

The morphological analysis was performed by TEM (FEI, TECNAI T12, operated at 120 kV) and SEM (Field Emission, Zeiss ΣIGMA, operated at 5–10 kV, 10 μm aperture).

### Gas sensing setup

The experimental setup for gas sensing measurements is reported in [23]. The MWCNT-based chemiresistors were placed in a 500 mL sealed stainless test cell for gas sensing measurements. In the cell, four gas sensors were simultaneously placed and exposed to the target gas flux. The reference gas was dry air (AirLiquide), used also as carrier during the exposure to dilute the target gas, maintaining a constant flow rate of 1000 sccm. Various software-controlled mass flowmeters (G-MIX, Bioage) at different full scales were used [19].

The gas sensing experiments were performed by evaluating the change in resistance of the active sensing layer in the two-pole format during exposure to the gaseous analytes, NO_2_ (0.1–10 ppm), H_2_S (0.1–10 ppm), NH_3_ (5–1000 ppm), C_4_H_10_ (5–1000 ppm), at operating temperatures in the range of 45–200 °C to evaluate the temperature effect on the gas sensing performance.

Each sensing cycle consisted of 60 min of stabilization, fixing the sensor signals under dry air flux, exposure for 10 min to the target gas at decreasing steps of concentration, and finally, 60 min of recovery to restore the initial sensor signal under dry air flux, after cleaning the test cell and active layer surface.

The response at a defined gas concentration was evaluated as the relative resistance variation in percentage, Δ*R*/*R*_i_ (%), where Δ*R* is the resistance variation between the values of the steady-state of the electrical resistance of the sensor under the target gas and under the reference/carrier gas (dry air), *R*_f_ and *R*_i_, respectively. The mean gas sensitivity, *S*_m_ (% ppm^−1^), is the weighted mean of the ratio between the relative resistance change in percentage, Δ*R*/*R*_i_ (%), over gas concentration unit (ppm). It can be calculated by the following equation:

[1]
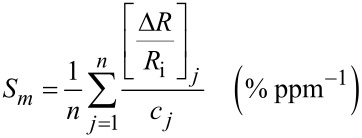


where Δ*R*/*R*_i_ (%) is the relative resistance change in percentage of the chemiresistor while exposed to the desired concentration of the target analyte *m*, *c_j_* (ppm) is the concentration value of the target analyte, and *n* is the number of gas exposures for each analyte.

## Results and Discussion

### Chemical and structural properties of metal-decorated MWCNTs

The elemental percentages detected by XPS on the surface of pristine and metal-decorated MWCNTs are reported in Table 1.

**Table 1:** XPS surface chemical composition of pristine and metal-modified MWCNTs.

Sample	C (at. %)	Au (at. %)	O (at. %)	O (at. %)/Au (at. %)

Pristine MWCNTs	95.0 ± 0.5	–	5.0 ± 0.5	–
Au NP/MWCNTs *t*: 90 s	94.4 ± 0.5	0.3 ± 0.2	5.3 ± 0.5	18
Au NP/MWCNTs *t*: 600 s	91.2 ± 0.5	1.1 ± 0.2	7.8 ± 0.5	7

Sample	C (at. %)	Pd (at. %)	O (at. %)	O (at. %)/Pd (at. %)

Pristine MWCNTs	98.5 ± 0.5	–	1.5 ± 0.5	–
Pd NP/MWCNTs *t*: 90 s	97.6 ± 0.5	0.3 ± 0.2	2.1 ± 0.5	7
Pd NP/MWCNTs *t*: 600 s	88.0 ± 0.5	1.5 ± 0.2	10.5 ± 0.5	7

As expected, the deposited metal content increases with the deposition time. The atomic percentage of oxygen at the MWCNT surface increases after the metal decoration, in both cases. Oxygen is commonly present in MWCNTs synthesized by the CVD process and is mainly derived from the oxygen adsorbed on the surface, as well as from carbon oxidation events. In both hybrid systems, the atomic percentage of oxygen further increased at the MWCNT surface after metal decoration, probably due to the generation of defects during the metal functionalization [40–41]. However, although the total oxygen content in the MWCNT films increased upon metal inclusion, the oxygen/gold elemental ratio decreased, while the oxygen/palladium ratio remained constant.

The chemical composition of the sensing layer at the interface, specifically the ratio between the surface oxygen content with respect to the surface metal loading, influences the interaction between the target gas and the sensing layer. This in turn controls the sensing properties of the metal-decorated MWCNTs, as reported in the following section.

The absence of XPS signals of N 1s, Cl 2p (in Au-modified MWCNTs), and Br 3d (in Pd-modified MWCNTs) derived from TOAC and TOAB surfactants, respectively, demonstrates (within the XPS limit-of-detection) the successful removal of the surfactant molecules from the metal surface after extensive device washing in ACN solution.

**Figure 3:**
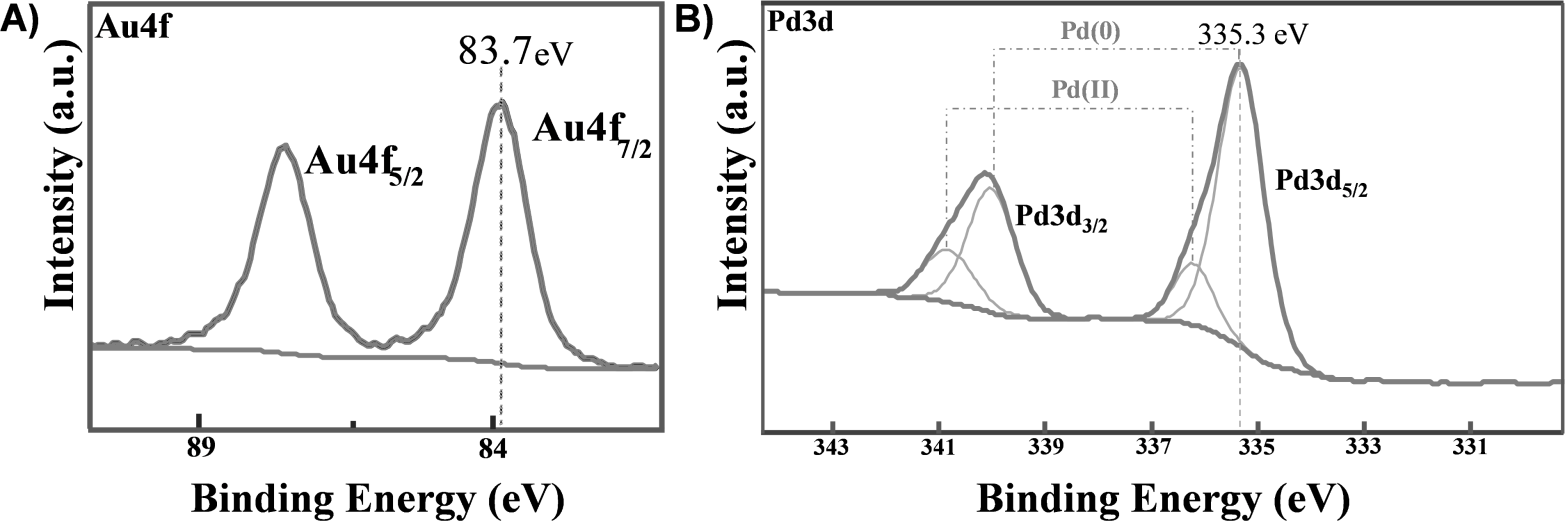
XPS core level spectrum of A) Au 4f and B) Pd 3d on functionalized MWCNTs.

**Figure 4:**
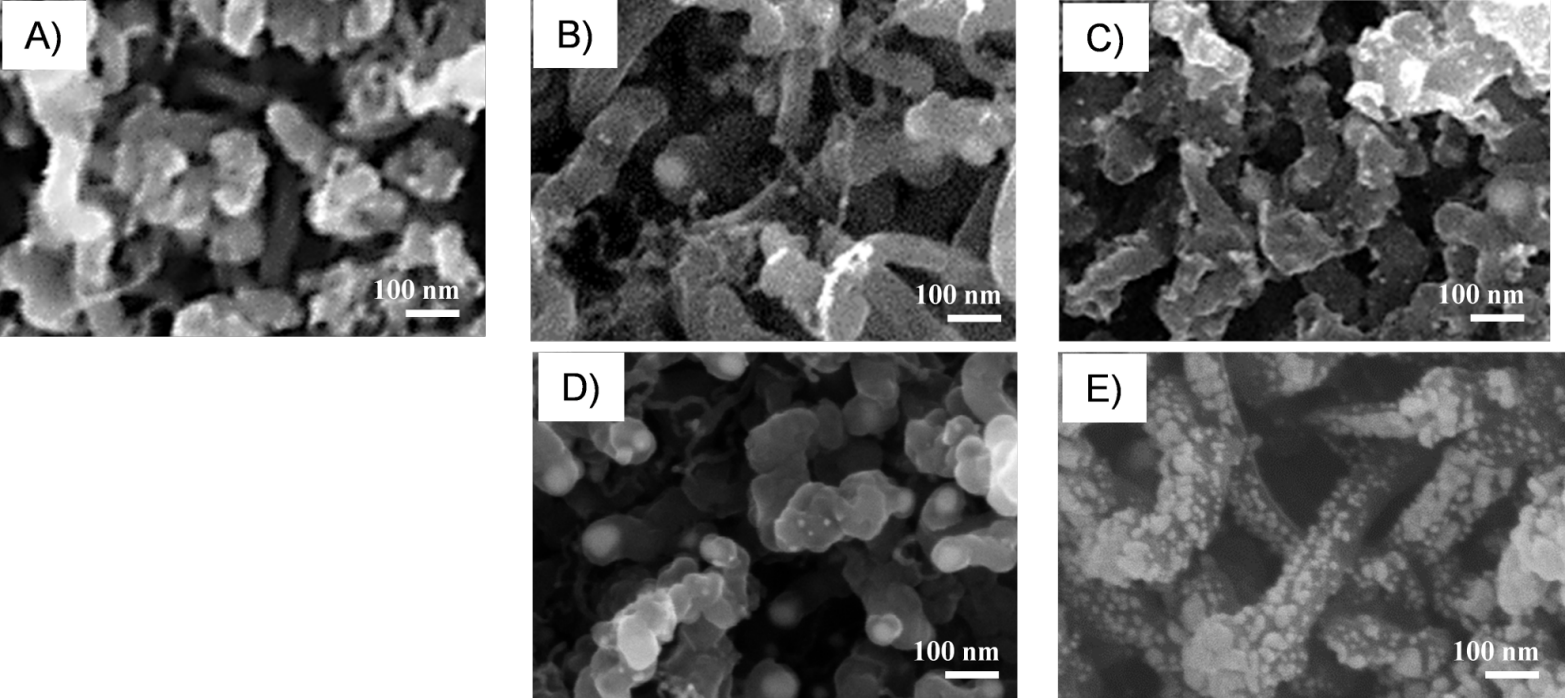
SEM images of A) pristine MWCNTs, and metal-decorated MWCNTs with B) 0.3 at. %, C) 1.1 at. % Au NP loading, and with D) 0.3 at. %, and E) 1.5 at. % of Pd NP loading.

Figure 3A, shows the Au 4f spectrum of Au-decorated MWCNTs, which clearly indicates a single doublet. This is attributed to nanometer-sized elemental gold according to the literature [19,42–43]. On the contrary, the Pd 3d XPS spectrum of Figure 3B is composed of two doublets and indicates the simultaneous presence of two chemical states. The most abundant chemical environment is attributed to elemental nanometer-sized palladium, and the other one to PdO [44]. Figure 4 reports the morphology of pristine and metal-decorated MWCNTs.

In all SEM images, networked tubes of nanometer dimension (10–30 nm in diameter, 300–500 nm in length) reveal the characteristic structure of MWCNTs. In functionalized MWCNTs, nanometer-sized metal particles partially decorate the surface of MWCNT sidewalls, with a higher density at higher metal loadings. These images confirm the control of the density of the deposited metal NPs by electrophoretic deposition time. Specifically, at a higher metal content, the Au NPs remain isolated, decorating the MWCNT sidewalls in a noncontinuous manner (Figure 4C). On the contrary, an almost contiguous distribution of Pd NPs covers MWCNT sidewalls as shown in Figure 4E.

In the following section, the effect of the nature and loading of the deposited catalytic metal NPs on the gas sensing properties of MWCNTs is discussed.

### Gas sensing properties

**Figure 5:**
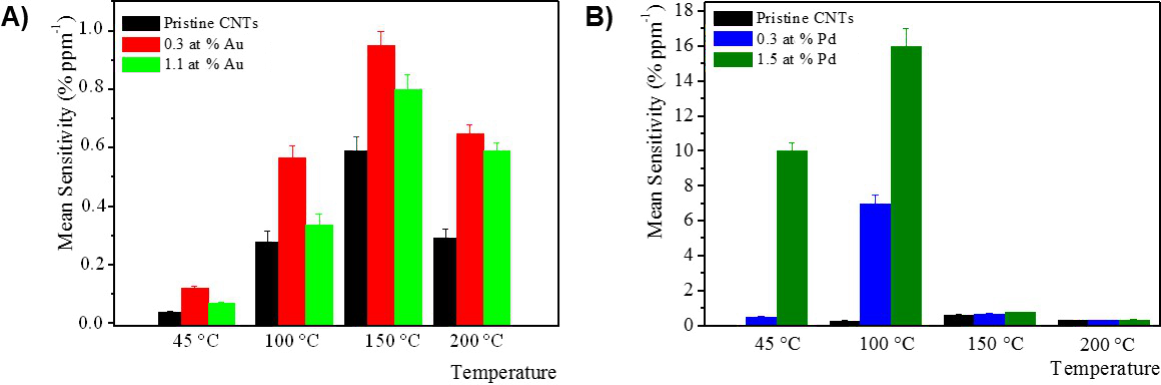
Mean sensitivity of pristine and A) Au- and B) Pd-modified MWCNTs-based sensors toward NO_2_ gas at different sensor operating temperatures in the range 45–200 °C.

The effect of the working temperature in the range 45–200 °C on NO_2_ detection is outlined in Figure 5, where the mean sensitivity of pristine and Au- and Pd-decorated MWCNT sensors is compared.

Although these results are related to NO_2_ gas exposure, they can also be extended to the other tested gases. This clearly indicates that each hybrid metal-decorated MWCNT device has an optimum operating temperature. In all reported cases, the mean sensitivity of metal-decorated MWCNTs is always higher than that of pristine MWCNTs, and the maximum mean sensitivity has been obtained at the intermediate temperatures of 150 °C and 100 °C for Au- and Pd-decorated MWCNTs, respectively, for all investigated gases.

Typicall, a high operating temperature (>400 °C) will reduce the catalytic properties of metal nanoparticles, as they tend to agglomerate in clusters of larger size, thus reducing their surface area, with a consequent decrease of the gas sensitivity. Moreover, MWCNTs could decompose at higher temperatures.

A low temperature (room temperature), on the other hand, limits a rapid and reversible desorption of the gas from the surface of metal-doped MWCNTs, increasing the recovery process.

Therefore, mild heating at 100–150 °C favors the desorption of the gaseous species from the surface of the sensing active layer so as to restore resistance to the initial baseline with a full recovery without loss of the catalytic effects of metal NPs [35,45].

Above the critical temperature, the desorption of gaseous molecules from the surface of MWCNTs is accelerated by the decrease of the thermal conductivity of MWCNTs [46]. This results in the consequent lowering of the energy barrier, and therefore, a decrease of the sensing response [47–48].

**Figure 6:**
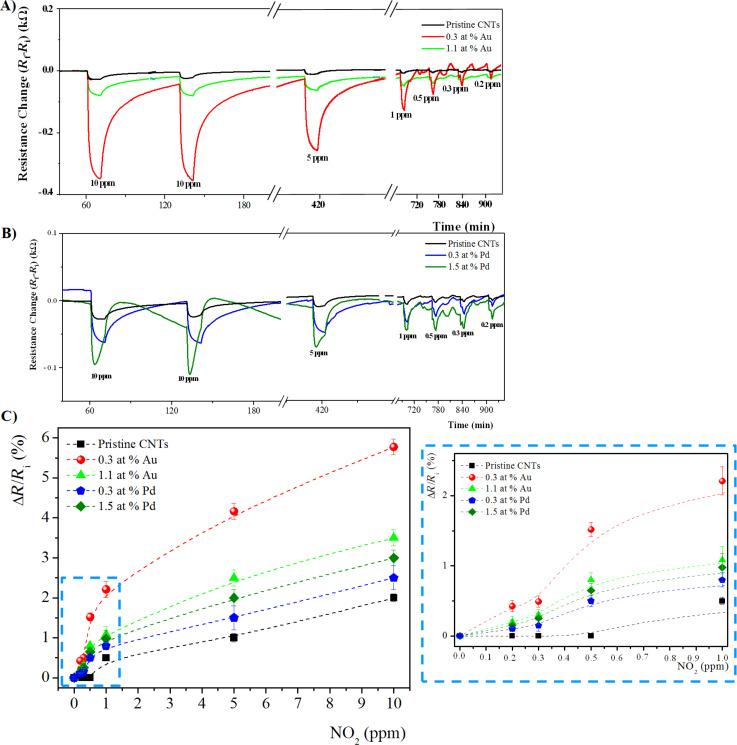
Time response of chemiresistors based on pristine and functionalized MWCNT films with A) Au loading of 0.3 at. %, and 1.1 at. %, and B) Pd loading of 0.3 at. % and 1.5 at. %, exposed to 10 min pulses of decreasing concentrations of NO_2_ at the sensor temperature of 150 °C. C) The corresponding calibration curves. The inset the plot is enlarged in the low concentration range (0.2–1 ppm).

**Figure 7:**
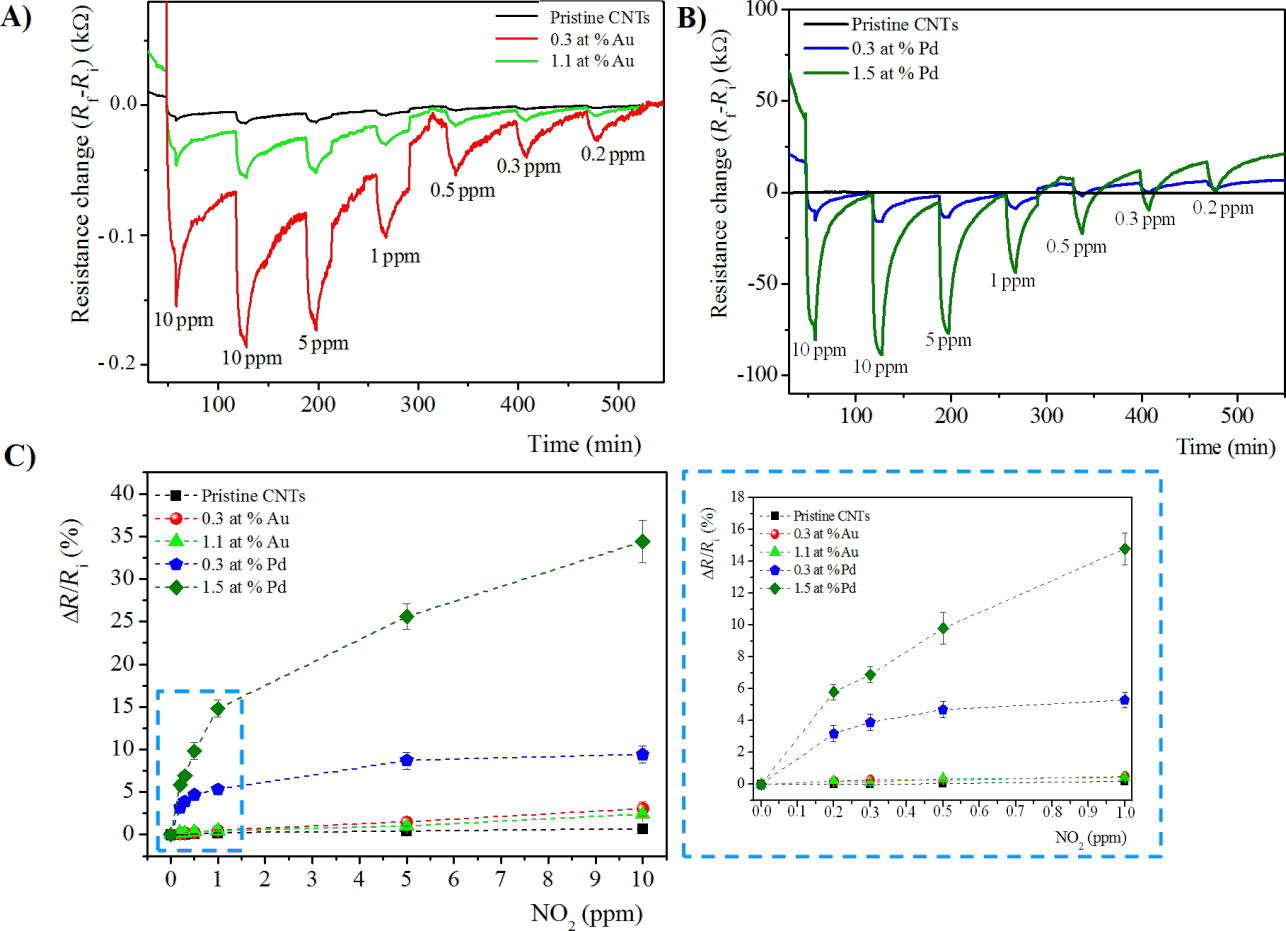
Time response of chemiresistors based on pristine and functionalized MWCNTs films with A) Au loading of 0.3 at. % and 1.1 at. %, and B) Pd loading of 0.3 at. % and 1.5 at. %, exposed to 10 min pulses of decreasing concentrations of NO_2_ at the sensor temperature of 100 °C. C) The corresponding calibration curves. The inset the plot is enlarged in the low concentration range (0.2–1 ppm).

Considering the specific optimum operating temperature of each hybrid sensing system, the sensing response, in terms of electrical resistance variation (Δ*R*), towards NO_2_ of pristine and metal-doped MWCNTs at 150 °C and 100 °C are reported in Figure 6A,B, and Figure 7A,B, respectively.

In all cases, at both operating temperatures, the sensing response, in terms of electrical resistance change (Δ*R*), of functionalized MWCNTs is higher compared to pristine MWCNTs, and increases with the gas concentration, as expected.

In the calibration plots of Figure 6C and Figure 7C, the sensor response is shown to change almost linearly up to about 1 ppm of NO_2_ while, above this value, the response variation is lower, probably due to the sensor saturation. Moreover, at both operating temperatures, the use of metal-modified MWCNTs led to higher responses to NO_2_, as compared to unmodified MWCNT films. In particular, at both operating temperatures, for the Au-modified MWCNTs, the active layer containing the lowest Au content (0.3 at. %) shows the highest response towards the target NO_2_ gas. Instead, for Pd-modified MWCNT networks, the sensing layer containing the maximum content of Pd NPs (1.5 at. %) exhibits the better response to NO_2_. Evaluating the effect of the metal at 100 °C, the Pd-functionalized MWCNTs show the highest response towards NO_2_. On the contrary, at 150 °C, the trend is opposite: Au-modified MWCNTs reveal the highest NO_2_ sensitivity.

**Figure 8:**
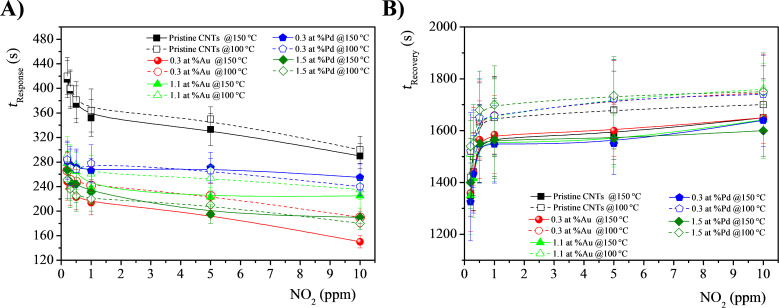
Variation of A) the response time (*t*_Response_) and B) the recovery time (*t*_Recovery_) of pristine, Au- and Pd-modified MWCNTs at various NO_2_ concentrations and operating temperatures (150 and 100 °C).

Metal-functionalized MWCNT-based gas sensors also showed faster response and recovery kinetics, as compared to pristine ones. Specifically, the response time was reduced by about 2–3 min in the NO_2_ concentration range 10–0.2 ppm. In Figure 8 the response and recovery times of pristine, and Au and Pd-modified MWCNTs at different NO_2_ concentrations and at the operating temperatures of 150 and 100 °C are reported.

The response time decreases with an increase in the gas concentration at all operating temperatures; in contrast, the recovery time reveals an opposite trend. This result is explainable considering that the presence of metal NPs on the surface of MWCNTs improves the adsorption of gaseous molecules and therefore the interaction at the interface in the response process. Moreover, this strong interaction kinetically hinders the gas desorption in the recovery process. Indeed, the higher operating temperature reduces the recovery time, favoring the desorption process, in all evaluated cases.

**Figure 9:**
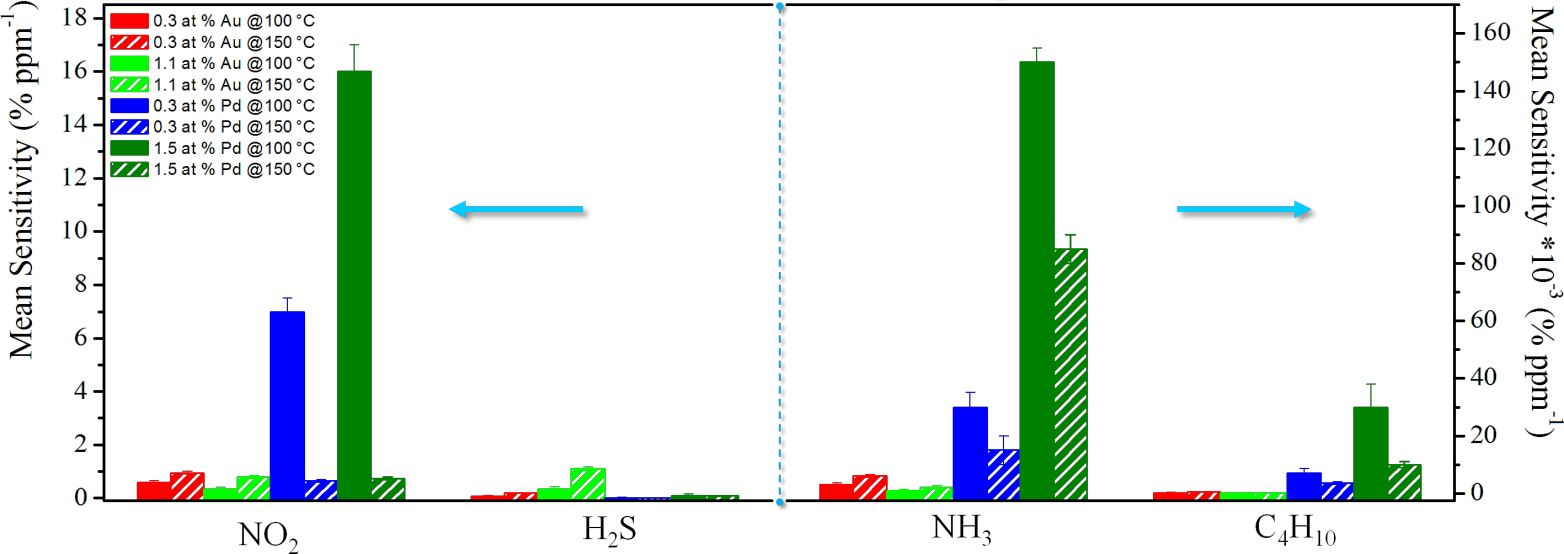
Comparison of mean sensitivity for four chemiresistors based on functionalized MWCTs with Au loading of 0.3 at. % and 1.1 at. %, and Pd loading of 0.3 at. % and 1.5 at. % exposed to NO_2_ [0.2–10 ppm], H_2_S [0.2–10 ppm], NH_3_ [5–1000 ppm] and C_4_H_10_ [5–1000 ppm] gases, at operating temperatures of 100 and 150 °C.

Considering the sensor operating-temperature effect on the sensing properties, Figure 9 compares the mean sensitivity of pristine and metal-functionalized MWCNTs towards NO_2_, H_2_S, NH_3_ and C_4_H_10_, at the both sensor temperatures, 100 and 150 °C.

The mean sensitivity of Pd-modified MWCNTs, specifically with the highest metal loading, towards NH_3_ and C_4_H_10_ was always higher than that of MWCNTs modified with Au NPs, at all operating temperatures. The highest mean sensitivity towards H_2_S was shown by Au-decorated MWCNTs at all sensing temperatures, especially for that with the highest Au content. The highest mean sensitivity towards NO_2_ was shown at 100 °C by Pd-modified MWCNTs, but at 150 °C by Au-functionalized MWCNTs.

Therefore, the gas sensitivity towards a specific gas is tailored by the type of metal used in the functionalization process, and by its loading deposited onto MWCNT networks. Therefore, it is also controlled by the operating temperature, which is specific for each system.

In particular, the MWCNT-based chemiresistors containing 0.3 at. % of Au shows the best response for NO_2_ at an operating temperature of 150 °C. On the other hand, at 100 °C, the MWCNTs functionalized with the highest Pd loading (1.5 at. %) revealed the best sensitivity towards NO_2_. The explanation of the higher NO_2_ gas sensing response obtained by MWCNTs functionalized with Au at the lowest content and with Pd at the highest loading could be given by the same sensing mechanism.

The evidence of a higher NO_2_ response from Au-modified MWCNTs with a low Au content has been already commented on and interpreted in [19] as being due not only to active Au nanophases, but also to oxygenated carbon species [41,49]. In fact, in the present case, Table 1 shows that at low Au loading, these oxygenated functionalities are higher.

For Pd-modified MWCNTs, the higher NO_2_ gas sensor response shown by MWCNT networks containing a high Pd loading could be explained by both chemical and electronic mechanisms, since in this case, as reported in Table 1, the oxygenated sites are preserved also at high metal content. Therefore, the high metal loading has a net total positive effect, improving the NO_2_ sensing.

Considering H_2_S gas sensing, it has already been demonstrated that a high gold content enhances the gas sensor response, thanks to the great affinity between gold and sulfur atoms of thiol groups [50]. Therefore, the increase in the Au NP density enhances the amount of the adsorption sites for H_2_S, resulting in a higher sensitivity and selectivity towards H_2_S gas.

Pd-functionalized MWCNTs have been shown to be highly sensitive to the other tested reducing gases, such as NH_3_ and C_4_H_10_. It is well known that Pd-modified CNTs are sensitive detectors for hydrogen and methane gases [51–53]. Therefore, the same proposed sensing mechanism can be extended, in this case, to explain the higher sensitivity of Pd-functionalized MWCNTs in the detection of ammonia and small hydrocarbon gas molecules like butane [54]. According to [54–55], when present on the MWCNT surface, Pd NPs form a weakly bonded complex between Pd atoms and adsorbed oxygen molecules at room temperature. This reasonably weak complex easily dissociates to form additional oxygen species, activating the reaction between NH_3_ or C_4_H_10_ and the adsorbed oxygen species. This therefore enhances the sensitivity of the Pd-modified MWCNT films.

Finally, the metal-decorated MWCNT-based chemiresistors prepared in this work show high sensing performance in comparison to previously reported results. Detailed comparisons are summarized and shown in Table 2.

**Table 2:** A comparison of resistive-type CNT-based sensors.

Material	Gas	Concentration	*T*	Sensitivity (%)	Ref.

WO_3_/MWCNTs	NO_2_	5 ppm	RT	14	[56]
SnO_2_/CNTs	NO_2_	1 ppm	RT	10	[57]
Au/CNTs	H_2_S	100 ppm	200 °C	10	[23]
Cu/SWCNTs	H_2_S	5 ppm	RT	10	[58]
Au/CNTs	NH_3_	1000 ppm	200 °C	0.07	[23]
CNT/SnO_2_	C_4_H_10_	0.1%	200 °C	32.56	[59]
Au/MWCNTs	NO_2_	5 ppm 1 ppm	150 °C 150 °C	4.2 1.5	[19]
Pd/MWCNTs	NO_2_	5 ppm 1 ppm	100 °C 100 °C	25 10	this work
Au/MWCNTs	H_2_S	5 ppm	150 °C	2.6	[19]
Pd/MWCNTs	NH_3_	1000 ppm	100 °C	32	this work
Pd/MWCNTs	C_4_H_10_	1000 ppm	100 °C	14	this work

## Conclusion

In conclusion, pre-synthesized colloidal Au and Pd NPs with defined size have been successfully deposited directly on MWCNT-based chemiresistive gas sensors by electrophoresis. This allowed for the controlled deposition of the metal content, preventing clustering formation.

The gas sensing results demonstrate that the performance (e.g., sensitivity and selectivity) of the MWCNT-based chemiresistors can be controlled by the surface catalyst used in the MWCNT functionalization (Au or Pd) with a defined size and surface concentration. The deposition of noble metal NPs (Au and Pd) on the surface of MWCNT networks considerably improved the gas sensitivity with respect to the unmodified system towards various pollutant gases (NO_2_, H_2_S, NH_3_, C_4_H_10_). The functionalization of MWCNT sidewalls with Au and Pd NPs enhances the gas sensitivity to the sub-ppm level for NO_2_ and H_2_S gases and imparts a certain degree of selectivity at the optimum sensor temperature (100 or 150 °C), depending on the type of deposited metal.

Therefore, the proposed electrophoretic method to functionalize the surface of a MWCNT gas senor with metal NPs is fast, scalable and versatile compared to the other proposed processes.

Moreover, these MWCNT-based solid-state chemiresistive gas sensors seem to be a complementary solution to the common analytical instrumentation for gas detection in environmental monitoring.
